# Immunobiological Activity of Synthetically Prepared Immunodominant Galactomannosides Structurally Mimicking *Aspergillus* Galactomannan

**DOI:** 10.3389/fimmu.2017.01273

**Published:** 2017-10-13

**Authors:** Ema Paulovičová, Lucia Paulovičová, Martin Hrubiško, Vadim B. Krylov, Dmitry A. Argunov, Nikolay E. Nifantiev

**Affiliations:** ^1^Cell Culture Laboratory, Department of Immunochemistry of Glycoconjugates, Center for Glycomics, Institute of Chemistry, Slovak Academy of Sciences, Bratislava, Slovakia; ^2^Department of Clinical Immunology and Allergy, Oncology Institute of St. Elisabeth, Bratislava, Slovakia; ^3^Laboratory of Glycoconjugate Chemistry, N.D. Zelinsky Institute of Organic Chemistry, Russian Academy of Sciences, Moscow, Russia

**Keywords:** *Aspergillus*, *Candida*, galactomannan, mannan, RAW 264.7, cytokines

## Abstract

The study is oriented at the *in vitro* evaluation of the immunobiological activity and efficacy of synthetically prepared isomeric pentasaccharides representing fragments of *Aspergillus fumigatus* cell-wall galactomannan and containing β-(1→5)-linked tetragalactofuranoside chain attached to O-6 (**GM-1**) or O-3 (**GM-2**) of a spacer-armed mannopyranoside residue. These compounds were studied as biotinylated conjugates which both demonstrated immunomodulatory activities on the RAW 264.7 cell line murine macrophages as *in vitro* innate immunity cell model. Immunobiological studies revealed time- and concentration-dependent efficient immunomodulation. The proliferation of RAW 264.7 macrophages was induced at higher concentration (100 µg/mL) of studied glycoconjugates and longer exposure (48 h), with more pronounced efficacy for **GM-1**. The increase of proliferation followed the previous increase of IL-2 production. The cytokine profile of the macrophages treated with the glycoconjugates was predominantly pro-inflammatory Th1 type with significant increase of TNFα, IL-6, and IL-12 release for both glycoconjugates. The RAW 264.7 macrophages production of free radicals was not significantly affected by glycoconjugates stimulation. The phagocytic activity of RAW 264.7 cells was reduced following **GM-1** treatment and was significantly increased after 24 h stimulation with **GM-2**, contrary to 48 h stimulation. Moreover, the synthetically prepared galactomannoside derivatives have been evaluated as efficient serodiagnostic antigens recognized by specific Ig isotypes, and significant presence of specific IgM antibodies in serum of patients suffering from vulvovaginitis was observed.

## Introduction

Throughout the past decades, the incidence of opportunistic systemic fungal infections has a significant rise due to increased numbers of immunocompromised adult and pediatric individuals. *Candida* species are the most common ubiquitous medically important opportunistic fungi followed by *Aspergillus* species (1–3). Invasive aspergillosis is the second most frequent systemic fungal infection with increasing incidence over the last 20 years (4). *Aspergillus* spp. principally affect the lungs causing the four main *Aspergillus*-related syndromes: (i) allergic bronchopulmonary aspergillosis, (ii) chronic necrotizing *Aspergillus* pneumonia (also termed chronic necrotizing pulmonary aspergillosis), (iii) aspergilloma, and (iv) invasive aspergillosis. Hematogenous dissemination of *Aspergillu*s beyond the lungs has been documented in patients who are severely immunocompromised (5). Within an 1 year period (from 2016 until present), the 1.47-fold increase in *Aspergillus* spp. in clinical isolates from upper airways and 1.8-fold increase in *Aspergillus* spp. clinical isolates from the lower respiratory tract have been documented. Moreover, the cutaneous aspergilli isolates increased 2.15 times within this period (raw data were obtained and analyzed with permission of MEDIREX Inc., HPL Mycology Labs., Slovakia).

Invasive aspergillosis has become the major cause of morbidity and mortality in immunocompromised patients with mortality rates as high as 90%. Almost 61% of patients with invasive aspergillosis have an underlying hematologic disease or have undergone bone marrow transplantation (6). Risk factors for the development of invasive aspergillosis include prolonged or repeated episodes of severe neutropenia, transplantation of solid organs or receipt of an allogenic stem cell transplant, grade III or IV graft-vs.-host disease, prolonged use of corticosteroid therapy, treatment with T-cell immunosuppressants, and inherited severe immunodeficiency (7–12). Etiological agent of more than 90% of invasive mycoses caused by *Aspergilli* is an *Aspergillus fumigatus* (*A. fumigatus*) (13). The treatment of invasive fungal infections failed in nearly 50% cases of invasive aspergillosis (14). Furthermore, resistant *Aspergillus* infections are frequently encountered in the antifungal drug-naïve patient as a result of increasing incidence of environmental *A. fumigatus* isolates harboring azole resistance mechanisms. *In vivo* selection of acquired resistance during medical treatment is increasingly more accounted in the patients with chronic forms of aspergillosis on the long-term azole treatment (15).

The anti-*Aspergillus* host immune defense is mediated by a complex of responses of the innate immune system phagocytic cells, resident alveolar macrophages, which ingest and kill *Aspergillus* conidia. Next, polymorphonuclear leukocytes destroy germinating *Aspergillus* hyphae, which escaped from macrophages. Neutrophils participate in the adaptive T helper cell response that in turn modulates antifungal activity, enhancing the phagocyte effector cell function (16). There are intensive efforts to design the effective model of subcellular fungal vaccines for either active or passive immunization in humans based on the dominant fungal cell-wall derived moieties (17–21).

In the vaccine model, different preparations of *A. fumigatus* antigens accelerated expansion of various CD4 T-cell subsets (22). The vaccinating potential of different *Aspergillus* antigens against invasive pulmonary aspergillosis, using antigen with the immunoadjuvant murine CpG oligodeoxynucleotide (CpG/Ag model) and dendritic cells model has been also studied (22).

Generally, the *A. fumigatus* cell wall is composed of a fibrillar skeleton made of β-(1→3)-glucan chains with 3,6-branches bound to chitin, galactomannan, and β-(1→3)/(1→4)-glucan, embedded in an amorphous alkali-soluble cement mainly composed of α-(1→3)-glucan and galactose polymers: galactomannan and galactosaminogalactan (23, 24).

Galactomannan is a hetero-polysaccharide composed of a mannan core and galactofuransyl side-chain found in the cell wall primarily of mold-like fungi especially in *Aspergillus* spp. and *Penicillium* spp. but is also found in other species of fungi. The backbone chain of *Aspergillus* galactomannan comprises the (1→2)/(1→6)-linked α-d-mannopyranosyl residues substituted at O-3 or O-6 by oligo-β-d-galactofuranosyl-containing sidechains connected mainly *via* (1→5)-links. Such β-d-Gal*f*-bearing chains are regarded as immunodominant epitopes, especially when they are (1→5)-linked (25, 26). The immunodominant epitopes are located in tetra- and hexasaccharides containing β-d-Gal*f*-(1→5)-β-Gal terminal groups (27). Kudoh et al. reported the presence of β-1,6-linked Gal*f* residues in addition to the β-1,5-linked Gal*f* residues in the O-linked and N-linked carbohydrate moieties of the galactomannan from *A. fumigatus* (28). As concerned content, the differences between conidia and hyphae alkali-soluble and insoluble fractions thereof have been reported. The exposition of galactomannan in an alkali-insoluble fraction of conidia has been higher, i.e., 26 vs. 5% present in hyphae (29).

**Figure 1:**
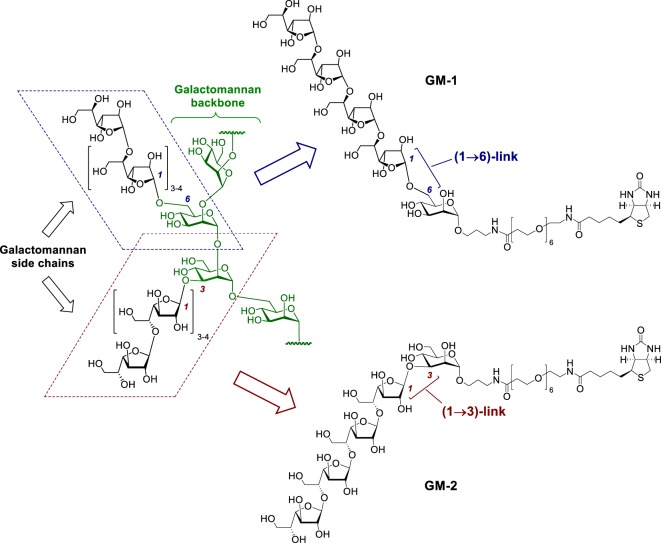
Hypothetical structure of the galactomannan secreted by *A. fumigatus* (**GM-native**) (25) and synthetic biotinylated probes **GM-1** and **GM-2** representing its (1→6)- and (1→3)-linked epitopes.

Herewith, we report for the first time a comparative study of the immunobiological activity and immunomodulating efficacy of synthetically prepared pentasaccharide derivatives **GM-1** and **GM-2** (Figure 1), whose structure mimics the corresponding fragments of *A. fumigatus* galactomannan bearing β-(1→5)-linked tetragalactofuranoside chain attached to O-3 or O-6 of the mannopyranoside residue. This study is focused on the assessment of proliferative activities, stimulated release of Th1and Th17 interleukins and growth factors, phagocytosis, free radicals release, expression of CD11b and F4/80 following RAW 264.7 macrophages GM-1 and GM-2 exposure. The evaluation of synthetically prepared oligosaccharide conjugates structurally related to *A. fumigatus* galactomannan as serodiagnostic immunogens for *in vitro* diagnostics of mycosis has been studied.

## Materials and Methods

### Synthesis of Biotinylated Glycoconjugates GM-1 and GM-2

Glycoconjugates **GM-1** and **GM-2** were prepared by the biotinylation of parent ligands (30–32) according to the previously described (33) biotinylation protocol.

### Cell Culture and Exposition

Stock solution and individual glycoconjugate doses were prepared aseptically using pre-sterilized disposable plasticwares and apyrogenic, sterile aqua pro injectione (Fresenius, Kabi Italia S.r.l., Verona). The solutions were filtered using a syringe with a 0.2-µm filter (Q-Max^®^Syringe filter) under sterile laminar flow conditions (Biohazard II, Esco). The safety cabinet was wiped down with 70% ethanol p.a. and sterilized with a germicidal UV lamp for 30 min before each experiment. The stock solution has been assayed with an EndoLISA^®^ enzyme-linked immunosorbent assay (ELISA)-based Endotoxin Detection Assay (Hyglos) and measured with a Cytation 5 Imager Multi-Mode Reader (BioTek, USA) to ascertain endotoxin-free conditions.

Cell line murine macrophages RAW 264.7 (ATCC^®^TIB-71™, ATCC, UK) were cultured in complete Dulbecco’s Modified Eagle Medium for 24 h at 37°C in a 5% CO_2_ atmosphere and 90–100% relative humidity until approx 80% confluency. Cell viability was assayed with the Trypan blue dye exclusion method using a TC20™ automated cell counter (Bio-Rad Laboratories, Inc., USA). The starting inocula of 1 × 10^5^ cells/mL/well (93.6% viable cells) were seeded in a 24-well cell culture plate (Sigma-Aldrich, USA) and exposed to 10 and 100 µg per well of **GM-1** and **GM-2** conjugates, respectively, to include two different concentrations with diverse stimulatory capabilities. Concanavalin A (Con A, 10 µg/mL, Sigma-Aldrich, St Louis, MO, USA), phytohemagglutinin (PHA, 10 µg/mL, Sigma-Aldrich, St Louis, MO, USA) and pokeweed mitogen (1 µg/mL, Sigma-Aldrich, St Louis, MO, USA) were used as positive controls.

*In vitro* exposition was performed for 3, 24, and 48 h, respectively. Morphological characteristics and viability were controlled ahead of flowcytometric evaluation. The exposed cells were subjected to immunocytometric determination of phagocytic activity and phenotyping immediately following cell separation by centrifugation. The cell culture media were stored at −20°C until further use.

### Proliferation and Cytotoxicity

The impact of **GM-1** and **GM-2** conjugates on RAW 264.7 cells’ proliferation and glycoconjugates’ potential cytotoxicity was evaluated by the bioluminescent measurement of adenosine triphosphate (ATP), marker of metabolically active cells levels, using the ViaLight™ plus kit (Lonza, USA) according to the manufacturer’s instructions. The intensity of emitted light was measured with a Cytation 5 Cell Imaging Multi-Mode Reader (BioTek Instruments, Inc., USA). Light emission expressed as relative light units was recorded continuously for 1 s and evaluated on the basis of peak values. The proliferation of unstimulated cells was considered to be the baseline. The proliferation index was calculated by the ratio between the induced proliferation (stimulated cells) and the baseline (unstimulated cells) proliferation. Hence, the proliferation index of negative control, i.e., unstimulated cells, is equal to one.

### Determination of Interleukins and Growth Factors

The levels of interleukins and growth factors in cell culture supernates mediated by the exposure with glycoconjugates **GM-1** and **GM-2**, respectively, were assayed with Quantikine ELISA^®^ Mouse M-CSF [Cat#MMC00, R&D, USA, minimum detectable dose (MDD) <5 pg/mL], Platinum ELISAs^®^: Mouse IL-12 (p70) (Cat#BMS616, MDD 4 pg/mL), Mouse GM-CSF (Cat#BMS612, MDD 2 pg/mL), Mouse IL-17(Cat#BMS6001, MDD 1.6 pg/mL), Mouse IL-2(Cat#BMS601, MDD 5.3 pg/mL), and Mouse IL-6 (Cat#BMS603/2, MDD 6.5 pg/mL); Instant ELISAs^®^: Mouse IL-1β (Cat#BMS600/2INST, MDD 3 pg/mL), Mouse tumor necrosis factor (TNF)-α (Cat#BMS607/2INST, MDD 4 pg/mL), Mouse IL-10 (Cat#BMS614INST, MDD 5.28 pg/mL), all from Affymetrix e-Bioscience, USA, according to the instructions of the manufacturer.

### Determination of Free Radicals

The cell culture supernates obtained after the treatment of RAW 264.7 cells by glycoconjugates **GM-1** and **GM-2** were assayed for total content of free radicals (Free radicals kit; SediumR&D, Czech Republic). The assay is based on the ability of chlorophyllin to transfer electrons due to its electron-rich double-bonds structure. The free radicals media levels were assayed *via* calibration based on a Fe^2+^/Fe^3+^ reactive shift and were expressed as millimoles Fe^2+^/L. The unstimulated cells free radical production was used to determine the baseline value.

### Immunocytometry

The **GM-1** and **GM-2** conjugates’ exposed RAW 264.7 cells were subjected to immunoflow cytometry using a Beckman Coulter FC 500 flow cytometer equipped with a 488-nm argon laser and a 637 nm HeNe collinear laser and controlled by the CXP software (Beckman Coulter, Fullerton, CA, USA). Gates were set to exclude the debris and damaged cells using forward scatter vs. side scatter dot plot discrimination. The settings were optimized either using proper isotype control (in immunophenotyping assay) or *Candida albicans* fluorescein isothiocyanate (FITC)-untreated cell culture (in phagocytosis). For each sample fluorescence histograms of 10,000 cells (immunophenotyping) or 5,000 cells (phagocytosis) were generated and analyzed (green fluorescence, 525-nm band-pass filter, FL1 channel). All samples were analyzed in duplicates. The data are expressed as percentage or as a mean of fluorescence intensity.

For immunocytometric assays, **GM-1** and **GM-2** conjugates exposed RAW 264.7 cells were stained directly with FITC-conjugated rat anti-mouse monoclonal antibodies: F4/80 and CD11b (both from eBioscience, Inc., CA, USA). The appropriate antibody isotype-negative controls were used separately to achieve correct gating. The FITC-conjugated monoclonal antibodies (5 µL) and **GM-1** and **GM-2** conjugates treated RAW 264.7 cells (50 µL) were added to 5-mL sterile tubes (Beckman Coulter, Fullerton, CA, USA) and incubated for 30 min in the dark at 4°C. After this, the samples were evaluated by immunoflowcytometry.

#### Phagocytosis

Measurement of phagocytosis, i.e., the ingestion of labeled *Candida albicans* (*C. albicans*) cells, took place under controlled conditions, using incubation with (FITC)-labeled *C. albicans* for 30 min at 37°C. Following treatment, the reaction was stopped by placing the samples on ice. Based on the difference between the resulting total amount of phagocyting cells and the amount of phagocyting cells following fluorescence quenching using Trypan Blue, the amounts of adherent extracellular and ingested intracellular *Candida* cells were determined.

#### Fluorescence Quenching Cytofluorometric Assay

The extracellular FITC-fluorescence has been quenched by 0.4% trypan blue dye (Sigma-Aldrich, USA). Immunocytometric analysis of trypan blue treated RAW 264.7 cells was performed following 30-min cell incubation in dark. Differentiation between attached and ingested *C. albicans*–FITC labeled cells was performed using the same protocol as previously described.

### Study Population

The serological assays were performed in a patient cohort comprising forty female participans (38.2 ± 8.4 years) with atopy and a history of recurrent vaginal mycosis (Dept. Clin. Immunol. and Allergy). Inhalant allergy was present in 63% of patients. The exclusion criteria were recent or ongoing antibiotic or immunosuppressive therapy. *Candida* spp., *Aspergillus* spp., and *Saccharomyces* spp. isolated from vaginal (94.42%) or cervical (5.58%) swabs undergone typing and identification (MEDIREX Inc., HPL Mycology Labs., Slovakia). Alyostal® Stallergenes Skin prick test (SPT), including *C. albicans* allergen (Alyostal R Stallergenes), was performed on the patients’ forearm according to the international and national guidelines. SPT was evaluated after 15–20 min and rated as positive if the wheal diameter was ≥3 mm and the negative control was negative.

### Control Group

Sixty-five female blood donors (National Blood Service, Slovak Republic) aged 18–56 years (average 35.9 ± 18.6) were enrolled as healthy control subjects.

### Sera Samples

All sera samples have been taken before the onset of antifungal and/or immunomodulative therapy, respectively. The sera samples for the determination of anti-oligogalactomannan and anti-mannan antibodies were collected and immediately stored at −70°C until the further use. The specimens were analyzed retrospectively and the results had no influence on therapeutic decisions.

### Determination of Anti-GM-1 and -GM-2 IgG, IgA, and IgM Isotypes

The ELISA for the determination of IgG, IgA, and IgM sera antibodies specific to studied galactomannosides has been developed by the modification of ELISA anti-*Candida* II based on *C. albicans* cell glycan antigens (Biogema, Slovakia). Synthetically prepared biotinylated oligogalactomannans (in 0.2 M TRIS-HCl buffer pH 7.0) were applied onto streptavidin coated microplates (Bioamat, SNC, Italy) (2 µg/mL, 200 µL/well) for 24 h at room temperature. After that, the plates were overcoated with 0.05 M carbonate–bicarbonate buffer (pH 9.5) with 0.025% Tween 20 and washed out. The plates were blocked with 1% BSA in 0.05 M carbonate–bicarbonate buffer. Sera samples have been examined for the **GM-1-** and **GM-2**-specific IgG, IgA, and IgM antibodies with peroxidase-labeled anti-human IgA, IgG, and IgM antibodies (KPL, USA). The plates were developed with 3,3′,5,5′-tetramethylbenzidine chromogenic substrate (Kem-En-Tec Diagnostics) and scanned at 450/630 nm (Microplate reader MRXII, Dynex, USA). According to the absence of appropriate international standards, the concentrations of different Ig isotypic antibodies were evaluated based on the calibration curve using internal standard, i.e., positive sera pool with an established value of 100 arbitrary units (U). The cut-off values were calculated according to blood donors’ IgG/IgM/IgA anti-**GM-1** and **GM-2** sera values (average + 3 SD). The patients’ results were expressed as calculated mean ± SEMs of two independent measurements. Anti-mannan IgG, IgA, and IgM antibodies were assayed as previously described (34).

### Ethics

The research protocol and the study have been approved by the Local Ethical Committee of the Oncology Institute of St. Elisabeth, Bratislava, Slovakia (15.12.2010). Written informed consent to participate in the experimental research study and for blood collection and subsequent laboratory examinations, in accordance with the principles in the Helsinki Declaration, was obtained from each patient prior to study enrollment. All patients were recruited from the outpatient department of the Department of Clinical Immunology and Allergy. Patient’s age, disease process, drug history, family history, and clinical signs and symptoms were documented at the first visit of Clinical Immunology and Allergy ambulance as a standard procedure.

### Statistical Analysis

The results of *in vitro* experiments with patient sera and RAW 264.7 cells were evaluated as mean values ± SD. Normality of data distribution was evaluated according to Shapiro–Wilk’s test at the 0.05 level of significance. Statistical comparison was performed using one-way ANOVA and *post hoc* Bonferroni’s tests. The results were significant if the differences equaled or exceeded the 95% confidence level (*P* < 0.05). Statistics was performed using the ORIGIN 7.5 PRO software (OriginLab Corporation, Northampton, MA, USA). Pearson’s correlation coefficient was used to compare the strength of the relationship between immunobiological variables.

## Results

### GM-1 and GM-2 Glycoconjugates Possess the Capability to Increase the Proliferation of Murine Macrophages RAW 264.7

**Figure 2:**
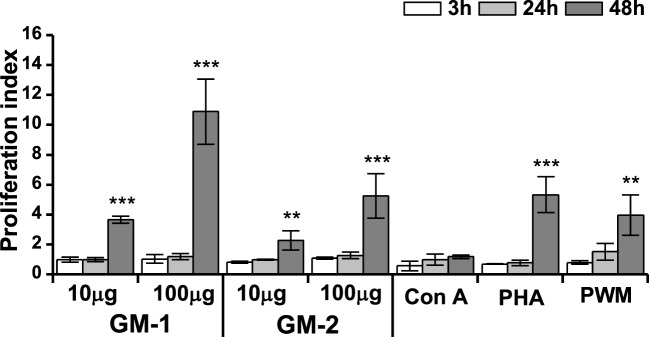
Effect of glycoconjugates GM-1 and GM-2 on proliferation of RAW264.7 macrophages. Cell proliferation was determined by ViaLight™ plus kit. RAW264.7 cells were treated with glycoconjugates **GM-1** or **GM-2** at concentrations of 10 and 100 µg/mL and with concanavalin A (Con A, 10 µg/mL), phytohemagglutinin (PHA, 10 µg/mL) and pokeweed mitogen (PWM, 1 µg/mL) as positive controls for 3, 24, and 48 h. Each value presents the mean ± SD of proliferation index (ratio of average relative light units (RLU) in the presence of stimulant to average relative light units obtained without stimulation). According to calculation formula of proliferation index, the proliferation index of negative control-untreated cells is equal to one. The statistical significance of differences between stimulated cells and untreated cells using one-way ANOVA and *post hoc* Bonferroni’s tests is expressed: **—0.001 < *P* < 0.01, ***—*P* < 0.001.

The effect of pentasaccharide-biotin conjugates **GM-1** and **GM-2** on the macrophage cell line RAW 264.7 proliferation was monitored by ATP bioluminescence as a marker of cell viability using the ViaLight™ plus kit. As shown in Figure 2, shorter stimulation periods (3 and 24 h) of RAW 264.7 cells did not alter the proliferation of macrophages. The 48-h stimulation resulted in a dose-dependent increase in RAW 264.7 proliferation for both tested biotinylated pentasaccharides. Thus, the stimulation with higher concentration of glycoconjugates (100 µg/mL) induced significantly more pronounced increase of RAW 264.7 proliferation (**GM-1:** 2.97-fold, and **GM-2:** 2.31-fold) compared to the 10 µg/mL concentration with even higher efficacy for glycoconjugate **GM-1** than **GM-2**.

### Cytokine and Free Radical Responses of RAW 264.7 Macrophages *In Vitro* to GM-1 and GM-2 Glycoconjugates

Generally, macrophages are tissue-resident professional phagocytes and antigen-presenting cells which differentiate from circulating peripheral blood monocytes. The diversity and plasticity is typical for the cells of the monocyte-macrophage lineage. The initial cross-talk and interaction of macrophages with specific cytokines following antigenic trigger determines their functional phenotype, thus influencing their engagement in processes of the antigen-presentation, phagocytosis, and regulatory functions. Microenvironment in which macrophages are situated provides diverse signals that could influenced the cell proliferation and differentiation, secretion of free radicals and is able to divergently bias the macrophage’s phenotype toward highly microbicidal or immunosuppressive macrophages.

**Figure 3:**
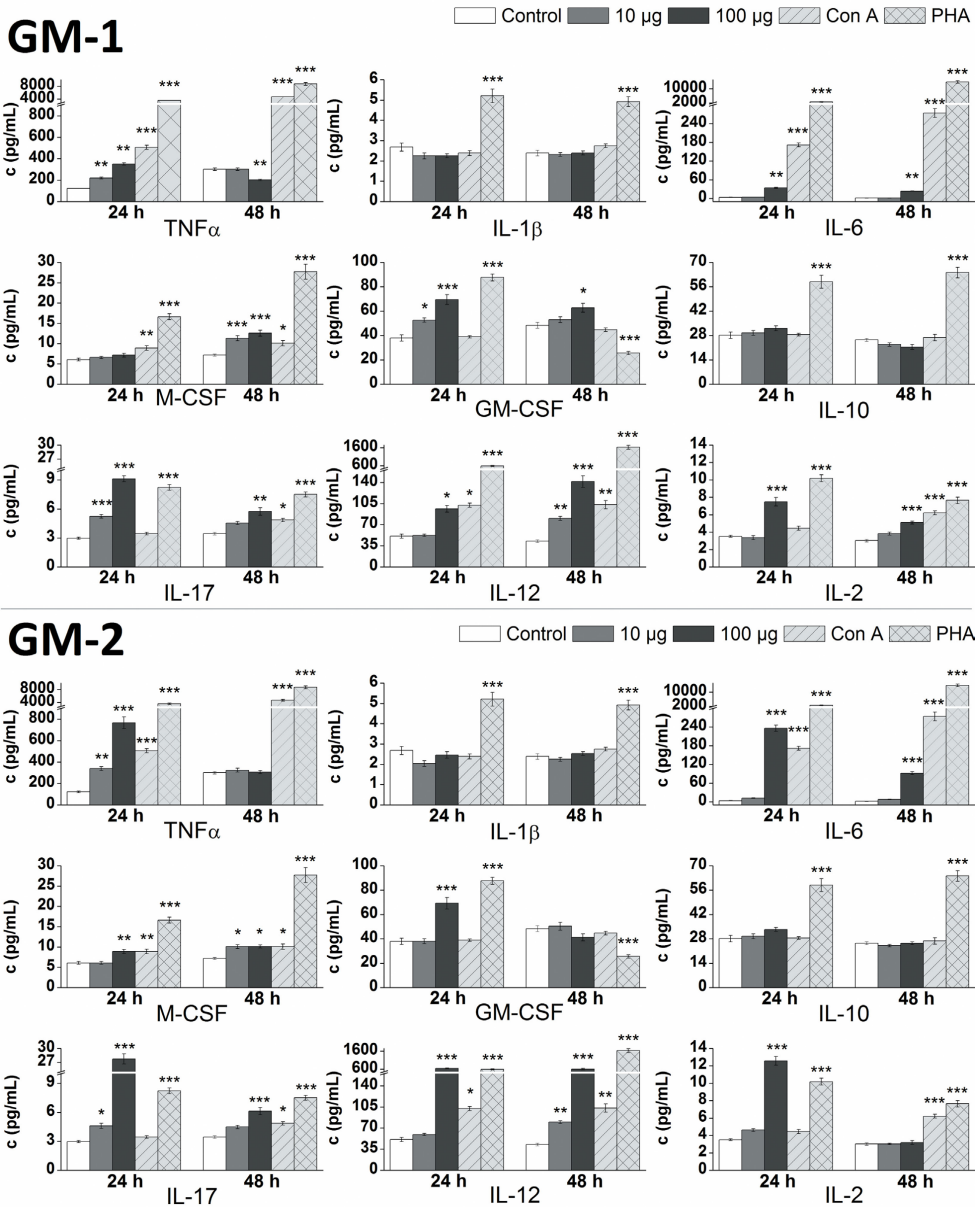
Effect of glycoconjugates GM-1 and GM-2 on RAW 264.7 macrophages cytokines production. Concentrations of cytokines in media after 24- or 48-h stimulation of RAW 264.7 macrophages in response to stimulation with 10 or 100 µg/mL concentration of glycoconjugates **GM-1** or **GM-2**, to positive controls concanavalin A (Con A, 10 µg/mL) and phytohemagglutinin (PHA, 10 µg/mL). Negative control represents untreated RAW 264.7 cells (Control). All data are presented as Mean ± SD. Tests were carried out in triplicate. The statistical significance of differences between stimulated cells and untreated cells using one-way ANOVA and *post hoc* Bonferroni’s tests is expressed: ***—*P* < 0.001, **—0.001 < *P* < 0.01, *—0.01 < *P* < 0.05.

The *in vitro* effect of glycoconjugates **GM-1** and **GM-2** on RAW 264.7 macrophages cytokines production was analyzed by the determination of pro-inflammatory cytokines TNFα, IL-1β, IL-6, IL-17, IL-12, IL-2, anti-inflammatory cytokine IL-10 and hemopoietic growth factors M-CSF, GM-CSF in supernatants obtained from cultures of RAW264.7 macrophages after the 24 or 48 h treatment (Figure 3). Stimulation of RAW 264.7 cells with glycoconjugate **GM-1** for 24 h resulted in a significant increase of TNFα (1.8-fold), IL-17 (1.8-fold), and GM-CSF (1.4-fold) production using a concentration of 10 µg/mL. Higher **GM-1** conjugate concentration (100 µg/mL) induced a more intense statistically significant increase of TNFα (2.9-fold), IL-17 (3.1-fold), and GM-CSF (1.8-fold) production and in addition increase of IL-6 (2.5-fold), IL-12 (1.9-fold), and IL-2 (2.1-fold), production during the 24-h treatment. Stimulation of RAW 264.7 cells with glycoconjugate **GM-1** for 48 h increased the M-CSF (1.9-fold) and IL-12 (1.9-fold) production by using concentration 10 µg/mL. The higher **GM-1** concentration (100 µg/mL) during 48-h stimulation induced a statistically significantly higher production, compared to the control, of mainly all analyzed cytokines except for pro-inflammatory cytokine TNFα (0.7-fold lower than control).

After the 24-h treatment at higher **GM-1** concentration (100 µg/mL) the production of pro-inflammatory cytokines TNFα, IL-6, IL-17, IL-12, IL-2, and hemopoietic growth factor GM-CSF were more efficiently induced compared to the lower **GM-1** concentration (10 µg/mL). The 48-h simulation period of RAW 264.7 cells determined the 100 µg/mL concentration of glycoconjugate **GM-1** as a preferable inducer of IL-6, IL-17, IL-12, IL-2, and both hemopoietic growth factors (M-CSF and GM-CSF) production (Figure 3). The stimulation of RAW 264.7 cells with glycoconjugate **GM-1** did not significantly influence the IL-1β and IL-10 production (Figure 3).

The 24-h stimulation period of RAW 264.7 cells with 10 µg/mL concentration of glycoconjugate **GM-2**, likewise GM-1 conjugate, significantly increased the production of pro-inflammatory cytokines TNFα (2.8-fold), and IL-17 (1.6-fold), but without effect on GM-CSF production. The higher **GM-2** concentration (100 µg/mL) possess similarly as GM-1 higher ability of cytokines induction and evoked an increase in the production of mainly all cytokines, with the highest effect on TNFα (6.3-fold), IL-6 (17.4-fold), IL-17 (9.3-fold), IL-12 (12.5-fold), and IL-2 (3.6-fold) production. After the longer 48-h stimulation period, the 10 µg/mL concentration of glycoconjugate **GM-2** increased the production of IL-6 (1.4-fold), M-CSF (1.4-fold), IL-17 (1.3-fold), and IL-12 (1.9-fold). Contrary to (1→6)-linked glycoconjugate **GM-1**, the 48-h stimulation of RAW 264.7 cells with (1→3)-linked isomer **GM-2** at 100 µg/mL concentration did not significantly increase the production of GM-CSF and IL-2, and induced a statistically significant increase of IL-6 (7.7-fold), M-CSF (1.4-fold), IL-17 (1.8-fold), and IL-12 (13.9-fold) production. Similarly as with glycoconjugate **GM-1**, the stimulation of RAW 264.7 cells with **GM-2** did not significantly influence the IL-1β and IL-10 production (Figure 3).

At higher concentration of 100 µg/mL, glycoconjugate **GM-2** more efficiently induced an increase in analyzed cytokines for both monitored time periods (24 and 48 h). The 10 µg/mL concentration of glycoconjugate **GM-2** evoked a time-dependent increase in cytokines production, favoring 48-h stimulation period. The 100 µg/mL concentration of **GM-2** more efficiently induced the production of cytokines during the 24-h stimulation time period (Figure 3).

**Figure 4:**
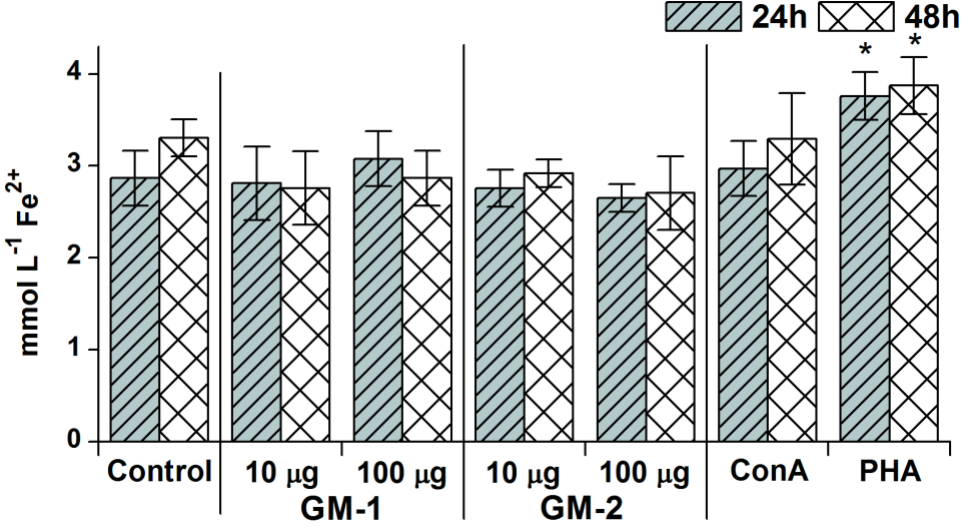
Concentration- and time-dependent pattern of free radicals’ release. The release after 24 or 48 h stimulation of RAW264.7 macrophages in response to stimulation with 10 or 100 µg/mL concentration of glycoconjugates **GM-1** or **GM-2** and to concanavalin A (Con A, 10 µg/mL) and phytohemagglutinin (PHA, 10 µg/mL) as a positive controls; control represents untreated cells (Control). All data are presented as mean ± SD. Tests were carried out in triplicate. The statistical significance of differences between stimulated cells and untreated cells using one-way ANOVA and *post hoc* Bonferroni’s tests is expressed: *—0.01 < *P* < 0.05.

Cell culture media following 24- and 48-h RAW 264.7 exposure with glycoconjugates **GM-1** and **GM-2** were assayed for free radicals release. The resulting values were compared with control (untreated cells) and with ConA or PHA treated cells (Figure 4).

Evidently, free radicals had been triggered only with a 100 µg/mL concentration of glycoconjugate **GM-1** following 24-h exposure; media release has been higher by 7% compared to untreated control. The concentration-dependent increasing trend has been observed for **GM-1** conjugate: 100 µg/mL concentration induces 10 and 4% increases in free radicals media levels following 24 and 48 h over the 10 µg/mL concentration induction, although the induced free radicals production did not significantly exceed the basal free radicals production of untreated RAW 264.7 cells. This tendency was not manifested with glycoconjugate **GM-2**. The overall inductive free radicals release caused by glycoconjugates **GM-1** and **GM-2** was apparently lower in comparison with ConA induction, except for 24-h treatment with glycoconjugate **GM-1** (100 µg/mL) resulting in 4% increase over the ConA induced level, and significantly lower than PHA induction of free radicals release (Figure 4).

### GM-1 and GM-2 Glycoconjugates Effect on the Phagocytic Activity of RAW264.7 Cells

The influence of glycoconjugates **GM-1** and **GM-2** on RAW 264.7 functionality has been evaluated on the basis of phagocytic capability. The phagocytic activity of exposed RAW264.7 cells and ingestion of *C. albicans*–FITC complex have been determined following 24- and 48-h exposure, respectively, with 10 and 100 µg/mL of both pentasaccharides **GM-1** and **GM-2**. To discriminate internalized FITC-labeled *Candida* cells from those attached to the cell membrane trypan blue, the quenching method has been applied as the cells attached to the cell membrane can be quenched (Table 1).

**Table 1:** RAW264.7 macrophage phagocytosis of *C. albicans*–FITC (%) following 24- and 48-h cell treatment with glycoconjugates GM-1 and GM-2 analyzed by flow cytometry.

Sample	Dose, μg/mL	Cell bound and internalized *C. albicans*–FITC		Internalized *C. albicans*–FITC		Membrane attached *C. albicans*–FITC	
		24 h	48 h	24 h	48 h	24 h	48 h
Control		39.3 ± 3.2	56.7 ± 4.2	12.1 ± 1.2	25.8 ± 2.1	27.2 ± 2.5	30.9 ± 1.8
GM-1	10	24.4 ± 2.3	27.4 ± 2.1**	4.8 ± 1.2**	1.6 ± 0.2***	19.6 ± 0.5*	25.8 ± 1.6
	100	21.7 ± 1.9	28.9 ± 1.7**	2.1 ± 0.9**	1.5 ± 0.3***	18.9 ± 0.9*	27.4 ± 0.9
GM-2	10	51.3 ± 3.4**	63 ± 2.9	7.7 ± 0.8	27.8 ± 2.5	43.6 ± 2.2**	35.2 ± 1.7
	100	46.4 ± 2.8*	56.7 ± 3.7	15.7 ± 1.8	24.9 ± 1.8	30.7 ± 1.9	31.8 ± 1.5

Control represents untreated cells (Control). The amount of membrane attached *C. albicans*–FITC is expressed as a difference between the population of cell membrane attached and cell internalized *C. albicans–*FITC and Trypan blue unquenched population, i.e., internalized cells. All data are presented as Mean ± SD. Tests were carried out in triplicate. The statistical significance of differences between untreated cells and stimulated cells using one-way ANOVA and *post hoc* Bonferroni’s tests is expressed as: ***—*P* < 0.001, **—0.001 < *P* < 0.01, *—0.01 < *P* < 0.05.

The immunocytometric assay of RAW 264.7 cells exposed to glycoconjugates **GM-1** and **GM-2** revealed the influence on effective phagocytosis of *C. albicans–*FITC complex. Evidently, both oligosaccharides exerted rather different effects especially on the process of cellular ingestion and internalization (Table 1). The amount of internalized *C. albicans–*FITC cells following the 48-h exposure of glycoconjugates **GM-1** at a concentration of 10 µg/mL resulted in a 16.1-fold decrease (*P* < 0.001) and following exposure with a concentration of 100 µg/mL the similar 17.2-fold decrease (*P* < 0.001) vs. untreated control has been determined. Compared to the results reached with glycoconjugate **GM-2**, the 17.4-fold decrease (at 10 μg/mL) and 16.6-fold (at 100 µg/mL) decrease have been detected after 48-h exposure. RAW 264.7 cells exposure to glycoconjugate **GM-2** did not regulate the ability of macrophage cells to phagocyte the *C. albicans–*FITC complex, and correlation analysis vs. control reveled *r* = 0.938 (10 µg/mL concentration) and *r* = 0.981 (100 µg/mL).

### The Influence of Conjugates GM-1 and GM-2 on RAW264.7 Macrophages Cell Surface Antigens F4/80 and CD11b Expression

**Figure 5:**
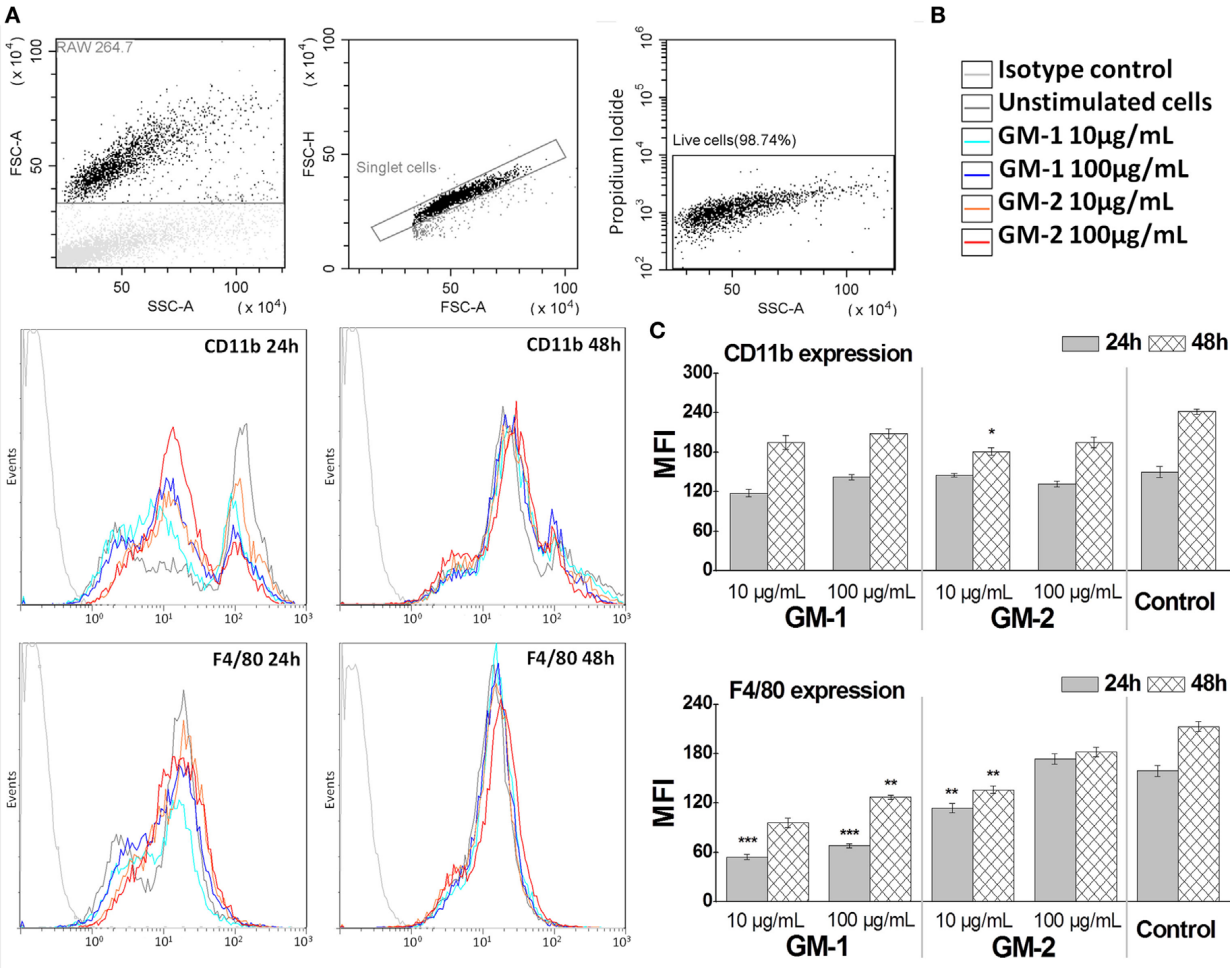
Immunocytometric measurement of RAW264.7 macrophage cell-line expression of major cell surface antigens F4/80 and CD11b following of the treatment by glycoconjugates GM-1 or GM-2 at 10 or 100 µg/mL concentrations. Gates were set to exclude the cellular debris using forward scatter vs. side scatter dot plot discrimination, doublets and dead cells **(A)**. The settings were optimized using proper isotype control. Fluorescence histograms **(B)** of 10,000 cells were generated and analyzed (green fluorescence, 525-nm band-pass filter). The data **(C)** are expressed as a mean of fluorescence intensity. The statistical significance of differences between untreated cells (Control) and stimulated cells using one-way ANOVA and *post hoc* Bonferroni’s tests is expressed as follows: ***—*P* < 0.001, **—0.001 < *P* < 0.01, *—0.01 < *P* < 0.05.

To establish the influence of conjugates **GM-1** and **GM-2** 24- and 48-h exposure on RAW264.7 macrophage cell-line, the macrophages major cell surface antigens F4/80 and CD11b (Mac-1 α; integrin αM chain part of the CD11b/CD18 heterodimer) expression has been followed (Figure 5). The treatment of RAW264.7 macrophage cells with glycoconjugates **GM-1** or **GM-2** at 10 or 100 µg/mL concentrations resulted in almost unchanged (24-h treatment) or a statistically insignificantly decreased (48-h treatment) CD11b expression, compared to non-treated control cells, except for significant decrease after 48-h treatment with glycoconjugate **GM-2** at 10 µg/mL concentration (33% decrease vs. control, *P* < 0.05). Comparing the individual conjugates in the context of the behavior of exposed RAW264.7 cells an increasing tendency of both surface antigens has been observed in the concentration- and time-dependent manner following the treatment with compounds **GM-1** or **GM-2** (Figure 5). The overall increase vs. control, i.e., untreated cells, has not been observed.

The kinetics of membrane protein F4/80 expression exerts the same trend; the expression of F4/80 is time- and concentration-dependent (Figure 5). The 24- and 48-h stimulation period of RAW 264.7 with 100 µg/mL concentration of glycoconjugate **GM-2** significantly increased the F4/80 expression over the expression triggered with 10 µg/mL (1.52-fold increase, *P* < 0.05). The decreasing trend of kinetics of F4/80 and CD11b following exposure with both structures, compared to control, i.e., untreated cells, has been more evident for isomer **GM-1** vs. control (2.96-fold for 10 µg/mL and 24 h, 2.2-fold for 100 µg/mL and 24 h vs. 1.39-fold and 1.17-fold for **GM-2** conjugate at the same time and concentration conditions).

The correlation between the time- and concentration-dependent CD11b and F4/80 expression on RAW264.7 macrophage cells following the exposure to (1→6)-linked isomer **GM-1** was *r* = 0.96 and while following the exposure to (1→3)-linked **GM-2** it was *r* = 0.46.

### Sera Levels of Anti-GM-1 and -GM-2 IgG, IgA, and IgM Isotypes

**Figure 6:**
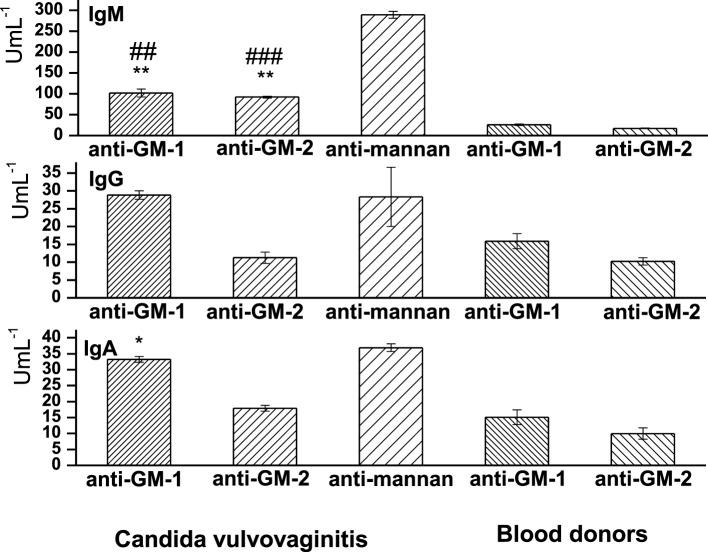
Distribution of sera levels of anti-GM-1, anti-GM-2, and anti-mannan isotype class antibodies in vulvovaginitis patients and healthy controls (blood donors). Data are presented as mean ± SD. Analyses were carried out in triplicate. The statistical significance of differences between controls (blood donors) and vulvovaginitis patients using one-way ANOVA and *post hoc* Bonferroni’s tests is expressed as: **—0.001 < *P* < 0.01, *—0.01 < *P* < 0.05. The statistical significance of differences between anti-mannan Abs and anti-**GM-1** and anti-**GM-2** Abs using one-way ANOVA and *post hoc* Bonferroni’s tests is expressed as follows: ^###^—*P* < 0.001, ^##^—0.001 < *P* < 0.01.

Sera levels of anti-**GM-1** and anti-**GM-2** antibodies were assayed in *Candida* vulvovaginitis patients and healthy controls (blood donors) (Figure 6). Evidently, the class distribution of anti-**GM-1** and anti-**GM-2** antibodies has revealed IgM as the highest abundant isotype, followed by IgA and IgG. Statistically, the most significant have been the sera levels of IgM anti-**GM-1** (*P* < 0.01) and IgM anti-**GM-2** (*P* < 0.01). The correlation between these two anti-pentasacchride specific IgMs is 0.99. The sera values of antigen specific IgM anti-**GM-1** were 3.92-fold increased vs. blood donors’ values; and for specific IgM anti-**GM-2** the 5.33-fold increase has been observed. Both pentasaccharide IgM isotypic antibodies were significantly lower in comparison with anti-*C. albicans* mannan antibodies, i.e., for IgM anti-**GM-2** (declined by 31.9%, *P* < 0.001, *r* = 0.96) and for IgM anti-**GM-1** the reduction was 35.22% (*P* < 0.01, *r* = 0.95).

The next most profound reactive isotype in the vulvovaginitis group has been IgA anti-**GM-1** demonstrated a 2.02-fold increase compared to healthy controls (*P* < 0.05). The 1.7-fold increase in specific IgA anti-**GM-2** over the healthy blood donors values has been statistically insignificant. The comparison with *Candida* mannan IgA response revealed a high correlation with anti-**GM-1** (*r* = 0.97) and a lower one for anti-**GM-2** (*r* = 0.81). Both pentasaccharides revealed similar IgA response compared to *C. albican*s mannan (Figure 6). The IgG antigen-specific response has been 1.81-fold increased with pentasaccharide **GM-1** over blood donors values and almost comparable with anti-mannan IgG (*r* = 0.98). The IgG anti-**GM-2** level was 1.09 times higher in the vulvovaginitis group than that in the control group. The vulvovaginitis group exerted a 2.52-fold decrease in anti-**GM-2** pentasaccharide IgG compared to anti-mannan IgG (*r* = 0.76).

## Discussion

The mold *A. fumigatus* is characterized by three morphotypes: (i) resting conidia, (ii) swollen conidia, and (iii) hyphae. The cell-walls of the mycelium and conidium are different especially at the level of the surface layer, which plays a significant role in the specific recognition of invading *A. fumigatus* by phagocytic cells of the immune system (24). The recognition of *A. fumigatus* conidia and hyphae is mediated by pattern recognition receptors (PRRs) either soluble and/or cell-bound receptors. The cell wall and its constituents symbolize the remarkable host–invader communication interface. Conidial germination starts with hydrophobic layer degradation and exposure of inner cell wall components, mainly polysaccharides such as chitin, β-glucan, mannan, and galactomannan. These represent pathogen-associated molecular patterns (PAMPs) recognized by PRRs (35, 36). Rizetto et al. (37) showed that the immune response induced by *Aspergillus* spp. may be dependent on variations of the fungus strain that could present diverse virulence factors and therefore increased or reduced infectivity. Generally, toll-like receptors and C-type lectin receptors as TLR2, TL4, and TLR9, Dectin-1, Dectin-2, DC-SIGN, mannose receptor, etc. on phagocytes directly recognize surface ligands on *A. fumigatus* and participate in pro-inflammatory and anti-inflammatory signaling responses resulting in cytokine and reactive oxygen species (ROS) release, thus supporting the antifungal activity (38–41). Of note, several polymorphisms of human TLRs, e.g., TLR1, TLR2, TLR4, TLR6, or TLR9, have been associated with increased risk of invassive aspergillosis in susceptible hosts (42–44).

Various interleukins and growth factors are engaged in host inter-reactivity with *A. fumigatus*. Bozza et al. (22) revealed that different preparations of *A. fumigatus* antigens induced the expansion of various CD4 T-cell subsets with secreted antigens promoting the differentiation of Th2 cells, membrane components Th1 cells, and glycolipids Th17 cells. In murine models of aspergillosis, α-(1→3)-glucan and β-(1→3)-glucan chains induce a protective response through the activation of Th1 and Th17 or Treg responses, whereas galactomannan favors the disease through the activation of the Th2/Th17 response (22).

In invasive aspergillosis, Th1-cell responses are associated with the resistance and onset of protective immunity, whereas Th2 responses are associated with progressive disease, more tissue damage, and poor survival. Th1-produced cytokines, including interferon-γ, interleukins IL-6, IL-12, TNF-α, and IL-1 activate neutrophils and pulmonary macrophages, the key effector cells in invasive aspergillosis, whereas Th2 cytokines, particularly IL-4 and IL-10, are associated with reduced IL-12 and TNF-α and worse outcome (45). Interestingly, the profile of the cytokine pattern depends on various aspects, such as the route of infection, immunological status of the host, type of *Aspergillus* antigens, etc. (2, 46, 47).

RAW 264.7 exposure to glycoconjugates **GM-1** and **GM-2** resulted in the accelerated cell-release of Th1 pro-inflammatory cytokines (Figure 3), TNF-α, IL-6, IL-12, IL-2, and hemopoietic growth factors GM-CSF and M-CSF associated with anti-*A. fumigatus* responses, this cytokine pattern is consistent with the results of Roilides et al. (48). Moreover, the G-CSF, GM-CSF, and M-CSF are cytokines with promising therapeutic efficacy and play critical roles in the host defense response during infection (49, 50). *In vitro* M-CSF has been shown to augment the antifungal activity of monocytes/macrophages against both conidia and hyphae of *A. fumigatus*, partly *via* enhancement of oxidation-dependent mechanisms (49).

Next, in a response to isomers **GM-1** and **GM-2** stimuli, the enhancement of media-release of IL-17 has been revealed. IL-17, signature cytokine of Th17 cells, is engaged in antimicrobial protection and induction of inflammation (51). Chai et al. has pointed out the role of this unique cytokine in Th17 anti-*A. fumigatus* immune responses (46). The Th1/Th17 polarized increased reactivity of the relevant signature cytokines TNF-a, IL-12, IL-17, IL-6, and IL-2 has been evidently structure-dependent, more apparent with (1→3)-linked **GM-2**, especially with 100 μg/mL concentration.

Resistance to *A. fumigatus* infection is associated with high levels of Th1 cytokines including IL-2, IL-12, and TNF-α (4). The statistically significant high media levels (*P* < 0.001) of these cytokines have been detected following the exposure with glycoconjugates **GM-1** and **GM-2** (Figure 3). On the contrary, disease progression is associated with Th2 cytokines IL-4 and IL-10 (4). Evidently, both galactomannosides **GM-1** and **GM-2** did not induce the significant IL-10 release over basal levels of untreated RAW2 64.7 cells (1.14-fold increase with 100 µg/mL of **GM-1** and 1.19-fold increase with 100 µg/mL of **GM-2**) (Figure 3).

Concerning concentration- and time-dependent cytokines’ release following cell exposure to glycoconjugate **GM-1**, the statistically significant tight correlations have been revealed between TNF-α and IL-1β (*r* = 0.96562, *P* = 0.03438); IL-6 (*r* = 0.99394, *P* = 0.00606); M-CSF (*r* = 0.99869, *P* = 0.00131), IL-12 (*r* = 0.99885, *P* = 0.00115); IL-2 (*r* = 0.92829, *P* = 0.04171); IL-17 (*r* = 0.94871, *P* = 0.04129); and IL-10 (*r* = –0.91585, *P* = 0.0325). Correlation analysis confirmed the similar trends of kinetics of these cytokines release also with isomer **GM-2**. The cell exposure to this compound (10 µg/mL) resulted in a tight correlation exerted by TNF-α and IL-1β (*r* = 0.87965, *P* = 0.012035); IL-6 (*r* = 0.99453, *P* = 0.00547); M-CSF (*r* = 0.99938, *P* = 0.000623); GM-CSF (*r* = 0.99291, *P* = 0.00709); IL-12 (*r* = 0.99968, *P* = 0.000315); IL-2 (*r* = 0.9579, *P* = 0.04205), IL-17 (*r* = 0.97497, *P* = 0.02503); and IL-10 (*r* = –0.90181, *P* = 0.0411).

Obviously, the Th1/Th17 prospectively antifungal protective immunobiological efficiency could be assumed. Conjugates **GM-1** and **GM-2** induced IL-10 tight negative correlation with TNF-α, associated with anti-inflammatory signaling are of interest. According to the observed time-dependent downregulation of several cytokines (from 24 to 48 h treatment), we can hypothesized engagement of relevant cytokine receptors and their interactions with media-produced cytokines subsequently resulting into the binding and uptake of released cytokine and regulatory feedback loop between cytokines and immune cells.

Thus, both synthetically prepared compounds **GM-1** and **GM-2** partially mimicking the *Aspergillus* galactomannan represented the appropriate model structures for *in vivo and in vitro* immunobiological studies. Their immunocompatibility has been confirmed based on the tests on functionality of RAW 264.7 macrophages treated with **GM-1** and **GM-2**, i.e., the trend of cell-line expression of major cell surface antigens F4/80 and CD11b following cell-exposure (Figure 5). Both markers represent pan macrophage antigens involved in cell adhesion and presumably in cell–cell interactions (F4/80) and, chemotaxis, phagocytosis, and apoptosis (CD11b) (Figure 5).

The induced changes of F4/80 expression demonstrated the association with concentration of glycoconjugates and exposition time as detected by cell-proliferation and phagocytosis. The free radical release did not exert this tendency (Figure 4).

The physiological concentration of ROS is essential to prevent immunometabolic disturbances. Oxidative stress induced by free radicals has been associated with the development of various diseases (52).

Obviously, phagocytosis plays the central role in anti-*Aspergillus* immunity. Innate effector phagocyting cells comprise alveolar macrophages, dendritic cells, neutrophils, and monocytes engaged in processes of hyphae and conidia engulfment, internalization, and killing *via* oxidative or non-oxidative mechanisms. *In vitro* studies documented the delay of conidial killing by alveolar macrophages after phagocytosis corresponding to the time when conidia become swollen (44). Interestingly, conidial phagocytosis involved DC-SIGN and complement recepor 3 and resulted in a protective Th1 response, while hyphal phagocytosis *via* Fc receptor and complement receptor 3 generates an unfavorable Th2 response (44). The experiments on RAW 264.7 phagocytosis and *Candida* internalization triggered by glycoconjugates **GM-1** and **GM-2** revealed a more efficient cell attachment and internalization with (1→3)-linked **GM-2** over (1→6)-linked **GM-1** (Table 1). Presumably, the structural epitopes of this formula resembled those of natural galactomannan more tightly compared to glycoconjugate **GM-1**. Evidently, both conjugates are able to initiate the process of phagocytosis, accompanied especially by the release of TNF-α, IL-6, IL-12, IL-2, Il-17 cytokines, and hemopoietic growth factors GM-CSF and M-CSF contributing to the regulation of inflammation (Figure 3). The significant increase of cell bound and internalized *C. albicans* cells by RAW 264.7 macrophages has been detected following 24 h cell pre-exposition with **GM-2** at 10 µg/mL (*P* < 0.01) and 100 µg/mL (*P* < 0.05).

Although the exposition of RAW 264.7 to **GM-1** upregulated the secretion of these cytokines and growth factors (Figure 3) to lesser degree than **GM-2**, the sequential process of phagocytosis has been inhibited (Table 1). Yet, we are not able fully to explain the decrease in phagocytosis despite the increase in Th1 type of cytokines. According to the known antigenic cross-reactivity between *Aspergillus* spp. and *Candida* spp. (53), one can suppose the interactions between *C. albicans* cellular PAMPs (e.g., mannan), **GM-1** and relevant RAW 264.7 PRRs. Evidently, **GM-1** comprised more cross-reactive epitopes reflecting the structure of candidal ones. This item needs further investigation.

Thus, the engagement of isomer (1→3)-linked **GM-2** in processes of recognition (recognition receptors DC-SIGN, Dectin-1, or TLRs) and subsequent attachment, engulfment and internalization is obviously over (1→6)-linked isomer **GM-1**. The DC-SIGN receptor binds dormant *Aspergillus* conidia in a galactomannan-dependent manner leading to the internalization of spores (54).

The commercial diagnostics of aspergillosis is based on EB-A2 monoclonal antibody reacting with the specific epitope of galactomannan. It is an IgM antibody with an avidity constant of 2 × 10^9^–5 × 10^9^ M binding to an epitope located on the β-(1→5) galactofuranosyl-containing side chain of the galactomannan molecule. The epitope recognized by the EB-A2 MAb is a common oligosaccharide moiety of a wide range of intracellular and extracellular glycoproteins of *Aspergillus* species EB-A2, and similar epitope seems to be present in other fungi (55, 56). Galactomannan is not unique to *Aspergillus* sp., apart from *Aspergillus* sp. galactomannan is found in different amounts also in *Penicillium, Fusarium, Alternaria*, and *Histoplasma* (57, 58). Moreover, Swanink et al. (53) suggested the reactivity of *Candida* sp. in galactomannnan assay due to cross-reacting antigens. In *Aspergillus*, histochemistry false positive staining of *Candida* was observed with both polyclonal and monoclonal *Aspergillus* antibodies (59). The serapositivity of anti-**GM-1** and -**GM-2** isotypic antibodies in *Candida*-colpitis cohort demonstrated the cross-reactivity of *Asperillus* related structures **GM-1** and **GM-2** (Figure 6). Evidently, these structures comprised the main cross-reactive epitopes.

For comparison, sera from vulvovaginitis patients have been subjected to seradiagnostics based on glycoconjugates **GM-1** and **GM-2**, in parallel with *Candida* mannan assay. The pattern of antigenspecific IgM > IgA > IgG immune responses reactive with structures **GM-1** and **GM-2** (Figure 6) reflected the previously observed kinetics of anti-*C. albicans* mannan and anti-*C. albicans* glucan antibodies in atopic patients suffering from vulvovaginitis (34), thus suggesting the immunobiological importance of such fungal oligoglycosidic structures. Our recent findings demonstrate the IgM as dominant isotype also in serological studies of Candida vulvovaginitis patients using different synthetically prepared glycosides (60). Generally, specific anti-glycoside IgM is elevated in active fungal infection along with IgA, while elevated antigen-specific IgG are characteristic for reccurent attacks (61). Nowadays, the specific role of natural IgM antibodies has been stressed (62, 63).

The *Aspergillus* gynecological infections are rare, according to MEDIREX Inc., HPL Mycology Labs., Slovakia, *Aspergillus* sp. represent 1/5,500 of all positive mycological isolates from uterus and cervix. Gupta et al. (64) reported simultaneous infection with *Aspergillus* sp. and cervical squamous cell carcinoma in the female genital tract attributed to the opportunistic nature of infection in the immunocompromised state due to the underlying malignancy. Genitourinary aspergillosis is rare in non-immunocompromised patients, only few reports have been documented (65–67).

## Conclusion

The ability of synthetically prepared isomeric galactomannoside derivatives **GM-1** and **GM-2** related to *Aspergillus* galactomannan antigen to interact with murine macrophages RAW 264.7 was studied. Significant immunomodulative effectivity of glycoconjugates **GM-1** and **GM-2** has been established *via* proliferation/cytotoxicity assay, phagocytosis and inductive interleukins and growth factors release. The protective Th1 and Th17 polarization has been revealed, especially with (1→3)-linked **GM-2**, which is more efficient trigger of the internalization and of *Candida* engulfment by RAW 264.7 cells.

The sera-crossreactivity with glycoconjugates **GM-1** and **GM-2** observed in vulvovaginitis patients revealed the clinical relevance of studied pentasccharide chains especially one with βGalf-(1→6)-αMan fragment present in the pentasaccharide **GM-1**.

In conclusion, it can be stated that synthetically prepared glycoconjugates **GM-1** and **GM-2** partially mimicking the structure of *Aspergillus* galactomannan represent the suitable *in vitro* and prospectively *in vivo* models for further immunobiological and immunotoxicological studies, potential antigens for *in vitro* diagnostics of aspergillosis and antifungal therapy monitoring.

## Ethics Statement

The research protocol and the study have been approved by the Local Ethical Committee of the Oncology Institute of St. Elisabeth, Bratislava, Slovakia. Written informed consent to participate in the research study and for blood collection and subsequent laboratory examinations, in accordance with the principles in the Helsinki Declaration, was obtained from each patient prior to study enrollment. Patient’s age, disease process, drug history, family history, and clinical signs and symptoms were documented at the first visit.

## Author Contributions

EP—executed the experimental design and performance of immunobiological studies, performed the human serodiagnostics, analyzed and interpreted the data. LP—performed immunobiological experiments and graphic data evaluation. MH—identified patient cohort, determined patient feasibility, and managed patient recruitment for the trial. VK and DA—performed the chemical syntheses of the oligosaccharide derivatives and analyzed the results. NN—planned the synthetic study, analyzed the results of synthetic part, and compared the data with contemporary literature.

## Conflict of Interest Statement

The authors declare that the research was conducted in the absence of any commercial or financial relationships that could be construed as a potential conflict of interest.

## References

[1] Lehrnbecher T Groll AH Invasive fungal infections in the pediatric population. Expert Rev Anti Infect Ther (2011) 9(3):275–8. 10.1586/eri.11.1 21417864

[2] Romani L. Cell mediated immunity to fungi: a reassessment. Med Mycol (2008) 46(6):515–29. 10.1080/13693780801971450 19180748

[3] Weaver CT Hatton RD Mangan PR Harrington LE. IL-17 family cytokines and the expanding diversity of effector T cell lineages. Annu Rev Immunol (2007) 25:821–52. 10.1146/annurev.immunol.25.022106.141557 17201677

[4] Chotirmall SH Al-Alawi M Mirkovic B Lavelle G Logan PM Greene CM *Aspergillus*-associated airway disease, inflammation, and the innate immune response. Biomed Res Int (2013) 2013:723129. 10.1155/2013/723129 23971044 PMC3736487

[5] Pagano L Caira M Valentini CG Posteraro B Fianchi L. Current therapeutic approaches to fungal infections in immunocompromised hematological patients. Blood Rev (2010) 24(2):51–61. 10.1016/j.blre.2009.11.003 20056300

[6] Gregg KS Kauffman CA Invasive aspergillosis: epidemiology, clinical aspects, and treatment. Semin Respir Crit Care Med (2015) 36(5):662–72. 10.1055/s-0035-1562893 26398533

[7] Baddley JW Andes DR Marr KA Kontoyiannis DP Alexander BD Kauffman CA Factors associated with mortality in transplant patients with invasive aspergillosis. Clin Infect Dis (2010) 50(12):1559–67. 10.1086/652768 20450350 PMC2874071

[8] Barchiesi F Mazzocato S Mazzanti S Gesuita R Skrami E Fiorentini A Invasive aspergillosis in liver transplant recipients: epidemiology, clinical characteristics, treatment, and outcomes in 116 cases. Liver Transpl (2015) 21(2):204–12. 10.1002/lt.24032 25348192

[9] Brown GD Denning DW Gow NA Levitz SM Netea MG White TC Hidden killers: human fungal infections. Sci Transl Med (2012) 4(165):165rv13 10.1126/scitranslmed.3004404 23253612

[10] Munoz P Ceron I Valerio M Palomo J Villa A Eworo A Invasive aspergillosis among heart transplant recipients: a 24-year perspective. J Heart Lung Transplant (2014) 33(3):278–88. 10.1016/j.healun.2013.11.003 24559945

[11] Saghrouni F Ben Youssef Y Gheith S Bouabid Z Ben Abdeljelil J Khammari I Twenty-nine cases of invasive aspergillosis in neutropenic patients. Med Mal Infect (2011) 41(12):657–62. 10.1016/j.medmal.2011.09.011 22036518

[12] Singh N Suarez JF Avery R Lass-Florl C Geltner C Pasqualotto AC Risk factors and outcomes in lung transplant recipients with nodular invasive pulmonary aspergillosis. J Infect (2013) 67(1):72–8. 10.1016/j.jinf.2013.03.013 23567625

[13] Kauffman CA. Fungal infections. Proc Am Thorac Soc (2006) 3(1):35–40. 10.1513/pats.200510-110JH 16493149

[14] Herbrecht R Fluckiger U Gachot B Ribaud P Thiebaut A Cordonnier C Treatment of invasive *Candida* and invasive *Aspergillus* infections in adult haematological patients. Eur J Cancer Suppl (2007) 5(2):49–59. 10.1016/j.ejcsup.2007.06.007

[15] Arendrup MC. Update on antifungal resistance in *Aspergillus* and *Candida*. Clin Microbiol Infect (2014) 20(Suppl 6):42–8. 10.1111/1469-0691.12513 24372701

[16] Nedel WL Kontoyiannis DR Pasqualotto AC Aspergillosis in patients treated with monoclonal antibodies. Rev Iberoam Micol (2009) 26(3):175–83. 10.1016/j.riam.2009.04.001 19635439

[17] Carvalho A Cunha C Iannitti RG Casagrande A Bistoni F Aversa F Host defense pathways against fungi: the basis for vaccines and immunotherapy. Front Microbiol (2012) 3:176. 10.3389/fmicb.2012.00176 22590466 PMC3349272

[18] Cutler JE Deepe GS Jr Klein BS. Advances in combating fungal diseases: vaccines on the threshold. Nat Rev Microbiol (2007) 5(1):13–28. 10.1038/nrmicro1537 17160002 PMC2214303

[19] Edwards JE Jr. Fungal cell wall vaccines: an update. J Med Microbiol (2012) 61(Pt 7):895–903. 10.1099/jmm.0.041665-0 22267544 PMC3542710

[20] Pikman R Ben-Ami R Immune modulators as adjuncts for the prevention and treatment of invasive fungal infections. Immunotherapy (2012) 4(12):1869–82. 10.2217/imt.12.127 23240754

[21] Vecchiarelli A Pericolini E Gabrielli E Pietrella D. New approaches in the development of a vaccine for mucosal candidiasis: progress and challenges. Front Microbiol (2012) 3:294. 10.3389/fmicb.2012.00294 22905033 PMC3417234

[22] Bozza S Clavaud C Giovannini G Fontaine T Beauvais A Sarfati J Immune sensing of *Aspergillus fumigatus* proteins, glycolipids, and polysaccharides and the impact on Th immunity and vaccination. J Immunol (2009) 183(4):2407–14. 10.4049/jimmunol.0900961 19625642

[23] Komarova BS Orekhova MV Tsvetkov YE Beau R Aimanianda V Latge JP Synthesis of a pentasaccharide and neoglycoconjugates related to fungal alpha-(1 – >3)-glucan and their use in the generation of antibodies to trace *Aspergillus fumigatus* cell wall. Chemistry (2015) 21(3):1029–35. 10.1002/chem.201404770 25376936

[24] Latge JP. 30 years of battling the cell wall. Med Mycol (2017) 55(1):4–9. 10.1093/mmy/myw076 27609562

[25] Latge JP Kobayashi H Debeaupuis JP Diaquin M Sarfati J Wieruszeski JM Chemical and immunological characterization of the extracellular galactomannan of *Aspergillus fumigatus*. Infect Immun (1994) 62(12):5424–33. 7960122 10.1128/iai.62.12.5424-5433.1994 PMC303284

[26] Latge JP Mouyna I Tekaia F Beauvais A Debeaupuis JP Nierman W. Specific molecular features in the organization and biosynthesis of the cell wall of *Aspergillus fumigatus*. Med Mycol (2005) 43(Suppl 1):S15–22. 10.1080/13693780400029155 16110787

[27] Leitao EA Bittencourt VC Haido RM Valente AP Peter-Katalinic J Letzel M Beta-galactofuranose-containing O-linked oligosaccharides present in the cell wall peptidogalactomannan of *Aspergillus fumigatus* contain immunodominant epitopes. Glycobiology (2003) 13(10):681–92. 10.1093/glycob/cwg089 12851285

[28] Kudoh A Okawa Y Shibata N. Significant structural change in both O- and N-linked carbohydrate moieties of the antigenic galactomannan from *Aspergillus fumigatus* grown under different culture conditions. Glycobiology (2015) 25(1):74–87. 10.1093/glycob/cwu091 25187160

[29] Mouyna I Fontaine T Cell wall of aspergillus fumigatus: a dynamic structure. In: Latgé J-P Steinbach WJ editors. Aspergillus fumigatus and Aspergillosis. American Society of Microbiology (2009). p. 169–83.

[30] Argunov DA Krylov VB Nifantiev NE. Convergent synthesis of isomeric heterosaccharides related to the fragments of galactomannan from *Aspergillus fumigatus*. Org Biomol Chem (2015) 13(11):3255–67. 10.1039/c4ob02634a 25643073

[31] Argunov DA Krylov VB Nifantiev NE The use of pyranoside-into-furanoside rearrangement and controlled O(5) – > O(6) benzoyl migration as the basis of a synthetic strategy to assemble (1 – >5)- and (1 – >6)-linked galactofuranosyl chains. Org Lett (2016) 18(21):5504–7. 10.1021/acs.orglett.6b02735 27759393

[32] Krylov VB Argunov DA Vinnitskiy DZ Verkhnyatskaya SA Gerbst AG Ustyuzhanina NE Pyranoside-into-furanoside rearrangement: new reaction in carbohydrate chemistry and its application in oligosaccharide synthesis. Chemistry (2014) 20(50):16516–22. 10.1002/chem.201405083 25319316

[33] Tsvetkov YE Burg-Roderfeld M Loers G Arda A Sukhova EV Khatuntseva EA Synthesis and molecular recognition studies of the HNK-1 trisaccharide and related oligosaccharides. The specificity of monoclonal anti-HNK-1 antibodies as assessed by surface plasmon resonance and STD NMR. J Am Chem Soc (2012) 134(1):426–35. 10.1021/ja2083015 22087768

[34] Paulovicova E Bujdakova H Chupacova J Paulovicova L Kertys P Hrubisko M. Humoral immune responses to *Candida albicans* complement receptor 3-related protein in the atopic subjects with vulvovaginal candidiasis. Novel sensitive marker for *Candida* infection. FEMS Yeast Res (2015) 15(2):fou001. 10.1093/femsyr/fou001 25673750

[35] Inoue M Shinohara ML Clustering of pattern recognition receptors for fungal detection. PLoS Pathog (2014) 10(2):e1003873 10.1371/journal.ppat.1003873 24586145 PMC3930597

[36] Netea MG Ferwerda G van der Graaf CA Van der Meer JW Kullberg BJ. Recognition of fungal pathogens by toll-like receptors. Curr Pharm Des (2006) 12(32):4195–201. 10.2174/138161206778743538 17100622

[37] Rizzetto L Giovannini G Bromley M Bowyer P Romani L Cavalieri D. Strain dependent variation of immune responses to *A. fumigatus*: definition of pathogenic species. PLoS One (2013) 8(2):e56651. 10.1371/journal.pone.0056651 23441211 PMC3575482

[38] Loures FV Rohm M Lee CK Santos E Wang JP Specht CA Recognition of *Aspergillus fumigatus* hyphae by human plasmacytoid dendritic cells is mediated by dectin-2 and results in formation of extracellular traps. PLoS Pathog (2015) 11(2):e1004643. 10.1371/journal.ppat.1004643 25659141 PMC4450068

[39] Mambula SS Sau K Henneke P Golenbock DT Levitz SM. Toll-like receptor (TLR) signaling in response to *Aspergillus fumigatus*. J Biol Chem (2002) 277(42):39320–6. 10.1074/jbc.M201683200 12171914

[40] Roeder A Kirschning CJ Rupec RA Schaller M Korting HC. Toll-like receptors and innate antifungal responses. Trends Microbiol (2004) 12(1):44–9. 10.1016/j.tim.2003.11.003 14700551

[41] Taylor PR Martinez-Pomares L Stacey M Lin HH Brown GD Gordon S. Macrophage receptors and immune recognition. Annu Rev Immunol (2005) 23:901–44. 10.1146/annurev.immunol.23.021704.115816 15771589

[42] Carvalho A Cunha C Carotti A Aloisi T Guarrera O Di Ianni M Polymorphisms in toll-like receptor genes and susceptibility to infections in allogeneic stem cell transplantation. Exp Hematol (2009) 37(9):1022–9. 10.1016/j.exphem.2009.06.004 19539691

[43] Chai LY Vonk AG Kullberg BJ Netea MG. Immune response to *Aspergillus fumigatus* in compromised hosts: from bedside to bench. Future Microbiol (2011) 6(1):73–83. 10.2217/fmb.10.158 21162637

[44] Park SJ Mehrad B Innate immunity to *Aspergillus* species. Clin Microbiol Rev (2009) 22(4):535–51. 10.1128/CMR.00014-09 19822887 PMC2772361

[45] Sambatakou H Pravica V Hutchinson IV Denning DW. Cytokine profiling of pulmonary aspergillosis. Int J Immunogenet (2006) 33(4):297–302. 10.1111/j.1744-313X.2006.00616.x 16893395

[46] Chai LY van de Veerdonk F Marijnissen RJ Cheng SC Khoo AL Hectors M Anti-*Aspergillus* human host defence relies on type 1 T helper (Th1), rather than type 17 T helper (Th17), cellular immunity. Immunology (2010) 130(1):46–54. 10.1111/j.1365-2567.2009.03211.x 20002791 PMC2855792

[47] Sales-Campos H Tonani L Cardoso CR Kress MR. The immune interplay between the host and the pathogen in *Aspergillus fumigatus* lung infection. Biomed Res Int (2013) 2013:693023. 10.1155/2013/693023 23984400 PMC3745895

[48] Roilides E Filioti J Gil-Lamaignere C Cytokines and the host defense against *Aspergillus fumigatus*. In: Kotb M Calandra T, editors. Cytokines and Chemokines in Infectious Diseases Handbook. Totowa, NJ: Humana Press (2003). p. 215–26.

[49] Kandalla PK Sarrazin S Molawi K Berruyer C Redelberger D Favel A M-CSF improves protection against bacterial and fungal infections after hematopoietic stem/progenitor cell transplantation. J Exp Med (2016) 213(11):2269–79. 10.1084/jem.20151975 27811055 PMC5068229

[50] Kasahara S Jhingran A Dhingra S Salem A Cramer RA Hohl TM. Role of granulocyte-macrophage colony-stimulating factor signaling in regulating neutrophil antifungal activity and the oxidative burst during respiratory fungal challenge. J Infect Dis (2016) 213(8):1289–98. 10.1093/infdis/jiw054 26908736 PMC4799674

[51] Veldhoen M. Interleukin 17 is a chief orchestrator of immunity. Nat Immunol (2017) 18(6):612–21. 10.1038/ni.3742 28518156

[52] Hybertson BM Gao B Bose SK McCord JM. Oxidative stress in health and disease: the therapeutic potential of Nrf2 activation. Mol Aspects Med (2011) 32(4–6):234–46. 10.1016/j.mam.2011.10.006 22020111

[53] Swanink CM Meis JF Rijs AJ Donnelly JP Verweij PE. Specificity of a sandwich enzyme-linked immunosorbent assay for detecting *Aspergillus* galactomannan. J Clin Microbiol (1997) 35(1):257–60. 8968919 10.1128/jcm.35.1.257-260.1997 PMC229550

[54] Serrano-Gomez D Dominguez-Soto A Ancochea J Jimenez-Heffernan JA Leal JA Corbi AL. Dendritic cell-specific intercellular adhesion molecule 3-grabbing nonintegrin mediates binding and internalization of *Aspergillus fumigatus* conidia by dendritic cells and macrophages. J Immunol (2004) 173(9):5635–43. 10.4049/jimmunol.173.9.5635 15494514

[55] Barton RC. Laboratory diagnosis of invasive aspergillosis: from diagnosis to prediction of outcome. Scientifica (Cairo) (2013) 2013:459405. 10.1155/2013/459405 24278780 PMC3820361

[56] Stynen D Sarfati J Goris A Prevost MC Lesourd M Kamphuis H Rat monoclonal antibodies against *Aspergillus* galactomannan. Infect Immun (1992) 60(6):2237–45. 1375195 10.1128/iai.60.6.2237-2245.1992 PMC257149

[57] Huang YT Hung CC Hsueh PR *Aspergillus* galactomannan antigenemia in *Penicilliosis marneffei*. AIDS (2007) 21(14):1990–1. 10.1097/QAD.0b013e3282eeb413 17721116

[58] Mikulska M Furfaro E Del Bono V Gualandi F Raiola AM Molinari MP Galactomannan testing might be useful for early diagnosis of fusariosis. Diagn Microbiol Infect Dis (2012) 72(4):367–9. 10.1016/j.diagmicrobio.2011.12.009 22280997

[59] Schuetz AN Cohen C. *Aspergillus* immunohistochemistry of culture-proven fungal tissue isolates shows high cross-reactivity. Appl Immunohistochem Mol Morphol (2009) 17(6):524–9. 10.1097/PAI.0b013e3181a38e05 19602969

[60] Krylov Vadim B Paulovičová L Paulovičová E Tsvetkov Yury E Nifantiev Nikolay E Recent advances in the synthesis of fungal antigenic oligosaccharides. Pure Appl Chem (2017) 89(7):885–98. 10.1515/pac-2016-1011

[61] Casadevall A Antibody immunity and invasive fungal infections. Infect Immun (1995) 63(11):4211–8. 7591049 10.1128/iai.63.11.4211-4218.1995 PMC173598

[62] Gronwall C Vas J Silverman GJ. Protective roles of natural IgM antibodies. Front Immunol (2012) 3:66. 10.3389/fimmu.2012.00066 22566947 PMC3341951

[63] Ehrenstein MR Notley CA. The importance of natural IgM: scavenger, protector and regulator. Nat Rev Immunol (2010) 10(11):778–86. 10.1038/nri2849 20948548

[64] Gupta P Goyal S Kaushal M. Concomitant *Aspergillus* species infection and squamous cell carcinoma diagnosed on Pap Smear. Turk Patoloji Derg (2016) 32(1):54–6. 10.5146/tjpath.2013.01198 24272933

[65] Baggish MS Ventolini G Vulvovaginal colonization by *Aspergillus* species in nonimmunocompromised women. J Gynecol Surg (2008) 24(2):55–60. 10.1089/gyn.2008.B-00242-1

[66] Agarwal N Seth A Kulshrestha V Kochar S Kriplani A Spontaneous vesicovaginal fistula caused by genitourinary aspergillosis. Int J Gynaecol Obstet (2009) 105(1):63–4. 10.1016/j.ijgo.2008.11.005 19081566

[67] Ventolini G *Aspergillus* and vaginal colonization. J Anc Dis Prev Rem (2014) 2:e115 10.4172/2329-8731.1000e115

